# Down-regulation of miRNA-106b inhibits growth of melanoma cells by promoting G1-phase cell cycle arrest and reactivation of p21/WAF1/Cip1 protein

**DOI:** 10.18632/oncotarget.2527

**Published:** 2014-09-25

**Authors:** Ram Prasad, Santosh K. Katiyar

**Affiliations:** ^1^ Department of Dermatology, University of Alabama at Birmingham, Birmingham, AL, USA; ^2^ Environmental Health Sciences, University of Alabama at Birmingham, Birmingham, AL, USA; ^3^ Comprehensive Cancer Center, University of Alabama at Birmingham, Birmingham, AL, USA; ^4^ Birmingham Veterans Affairs Medical Center, Birmingham, AL, USA

**Keywords:** Melanoma, MicroRNA-106b, cell cycle, tumor xenograft growth, grape seed proanthocyanidins

## Abstract

MiR-106b is overexpressed in various types of cancers and is associated with the regulation of the carcinogenic processes. Using RT-PCR, we have identified overexpression of miRNA-106b in various melanoma cell lines (A375, Hs294t, SK-Mel28, SK-Mel 119, Mel 1241, Mel 1011 and Mel 928) as compared to its expression in normal human epidermal melanocytes (NHEM). The overexpression of miR-106b in melanoma cells (A375, Hs294t) was associated with greater cell proliferation capacity than NHEM. Treatment of A375 and Hs294t cells with anti-miR-106b resulted in inhibition of cell proliferation as well as G1-phase arrest. We determined the effects of grape seed proanthocyanidins (GSPs) on the expression of miRNA-106b and its underlying molecular targets. Treatment of A375 and Hs294t cells with GSPs resulted in suppression of the levels of miRNA-106b, cytotoxicity, G1-phase arrest and reactivation of p21/WAF1/Cip1. Dietary GSPs significantly inhibited growth of A375 melanoma cell tumor xenografts in nude mice, which was associated with reduction in the levels of miRNA-106b, tumor cell proliferation and increases in the levels of p21/WAF1/Cip1 protein. These studies suggest that miRNA-106b plays a crucial role in melanoma growth and that GSPs act as an inhibitor of miR-106b thereby blocking melanoma growth *in vitro* and *in vivo* models.

## INTRODUCTION

Malignant melanoma is an aggressive and deadly skin cancer that causes the majority of skin cancer-related deaths [1, 2]. Importantly, the incidence of melanoma is increasing rapidly in children [3]. According to a National Cancer Institute report, there were an estimated 76,700 new cases of melanoma and 9,710 melanoma-related deaths in the United States in 2013 [4]. Although efforts have been made to develop an understanding of the causes of melanoma progression and more effective therapies, they have met with limited success. As melanoma is a highly malignant cancer, an approach that reduces its growth and progression potential may facilitate the development of an effective strategy for its prevention or treatment.

MicroRNAs (miRNAs) are small noncoding RNA molecules that regulate gene expression by binding to the 3′-untranslated regions (3′UTRs) of specific mRNAs. Alterations in the expression level of miRNA are correlated with cancer development [5], and play an important role in cell proliferation, cell differentiation, and cell death. Many of these miRNAs possess oncogenic or tumor suppressor activity in various tumors [5, 6], but little is known about their potential role in melanoma progression. miR-106b is involved in multiple cancer/tumor types, such as gastric, hepatocellular, laryngeal, prostate, breast, endometrial, pancreas and gastric, and thyroid cancers, non-melanoma skin cancer and melanoma [7-15]. Recently, it has been reported that pRB/E2F and p21/WAF1/Cip1, which promote cell cycle progression, are direct targets of miR-106b [7, 16]. Cell-cycle progression relies on the activation of cyclins and cyclin-dependent kinases (CDKs), which act together in G1 phase to initiate S phase and in G2 phase to initiate mitosis. These kinases promote expression of cell cycle genes controlled by E2F transcription factors [17]. In addition, members of the retinoblastoma protein (pRb) family inhibit cell cycle entry through repression of E2F-regulated cell cycle genes [18]. When activated in the G1 phase, cyclin D/CDK4, 6 and cyclin E/CDK2 kinases phosphorylate pRb, thereby preventing its association with E2F and allowing E2F transcription factors to induce S-phase gene expression [18].

As phytochemicals are emerging new options for the prevention and treatment of melanoma [19], the proanthocyanidins from grape seeds (GSPs) were tested for their efficacy against melanoma and in particularly as an inhibitor of miR-106b. Grape seeds are rich in proanthocyanidins (60–70%), which are mainly composed of dimers, trimers, tetramers and oligomers of monomeric catechins or epicatechins [20, 21]. GSPs have been shown to have cytotoxic effects on tumor cells without having adverse effects on normal cells [22]. As is the case for other bioactive phytochemicals, GSPs have been shown to have anti-carcinogenic effects in some animal tumor models with no apparent signs of toxicity in animals [23-25]. In this study, we first examined the role of miRNA-106b on the progression of melanoma cells. We then evaluated the chemotherapeutic effect of GSPs in terms of the proliferative potential of melanoma cancer cells and whether it is mediated through their effects on miRNA-106b. For this purpose, we used various human melanoma cancer cell lines as an *in vitro* model, and ascertained whether GSPs inhibit the growth of melanoma cancer cells through its inhibitory effect on miRNA-106b expression. We present evidence that GSPs inhibit melanoma cancer cell proliferation and *in vivo* tumor xenograft growth and that they do so through: (i) down-regulation of miRNA-106b expression, and (ii) blocking of melanoma cell division in the G1 phase of the cell cycle through reactivation of tumor suppressor protein p21/WAF1/Cip1.

## RESULTS

### Overexpression of miR-106b in melanoma cell lines and its association with cell proliferation

**Figure 1:**
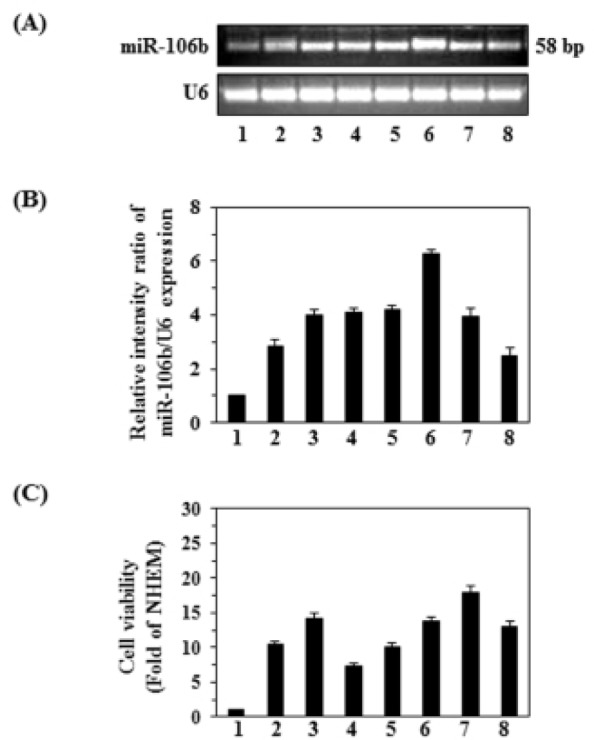
Comparison of the viability and expression of miR-106b in various melanoma cell lines with that of normal human epidermal melanocytes (NHEMs) (A) miRNAs from NHEMs and different melanoma cell lines were isolated and cDNA was subjected to RT-PCR. U6 was used as a loading control. (B) Relative band intensity of miR-106b expression in NHEM and different melanoma cell lines, mean values ±SD, n=2. (C) Cell viability assay revealed that the upregulation of miR-106b in melanoma cells was associated with greater cell proliferation. Cell viability was determined using an MTT assay and is expressed in terms of fold-change compared to NHEM control, n=5. Cell lines are assigned as: 1, NHEM; 2, A375; 3, Hs294t; 4, SK-Mel 28; 5, SK-Mel 119; 6, Mel 1241; 7, Mel 1011; and 8, Mel 928.

To explore the expression levels of miR-106b in human melanoma cell lines and normal human epidermal melanocytes (NHEM), we examined several human melanoma cell lines (A375, Hs294t, SK-Mel 28, SK-Mel 119, Mel 1241, Mel 1011, and Mel 928) as well as NHEMs using RT-PCR. As shown in Figure 1A, the melanoma cell lines express higher levels of miR-106b than NHEMs (amplicon size 58bp). The levels of miRNA-106b varied among the cell lines, with the highest amounts being found in the Mel 1241, SK Mel 119, SK Mel 28, Hs294t and Mel 1011 lines. In general, the expression levels of miRNA-106b in these cells lines is approximately 3- to 6-fold higher than in NHEMs, as estimated by densitometry quantification of the band intensity using imageJ software and calculation of the relative band intensity ratio of miR-106b *vs.* U6 (Fig. 1B). To assess the role of miR-106b on the progression of melanoma cells, we examined and compared the proliferating potential of various melanoma cell lines using an MTT assay. As shown in Figure 1C, overexpression of miR-106b in melanoma cell lines was associated with greater cell viability or proliferation potential, as is evident from the results shown in Figure 1B and Figure 1C.

### Suppression of miR-106b inhibits cell proliferation

**Figure 2:**
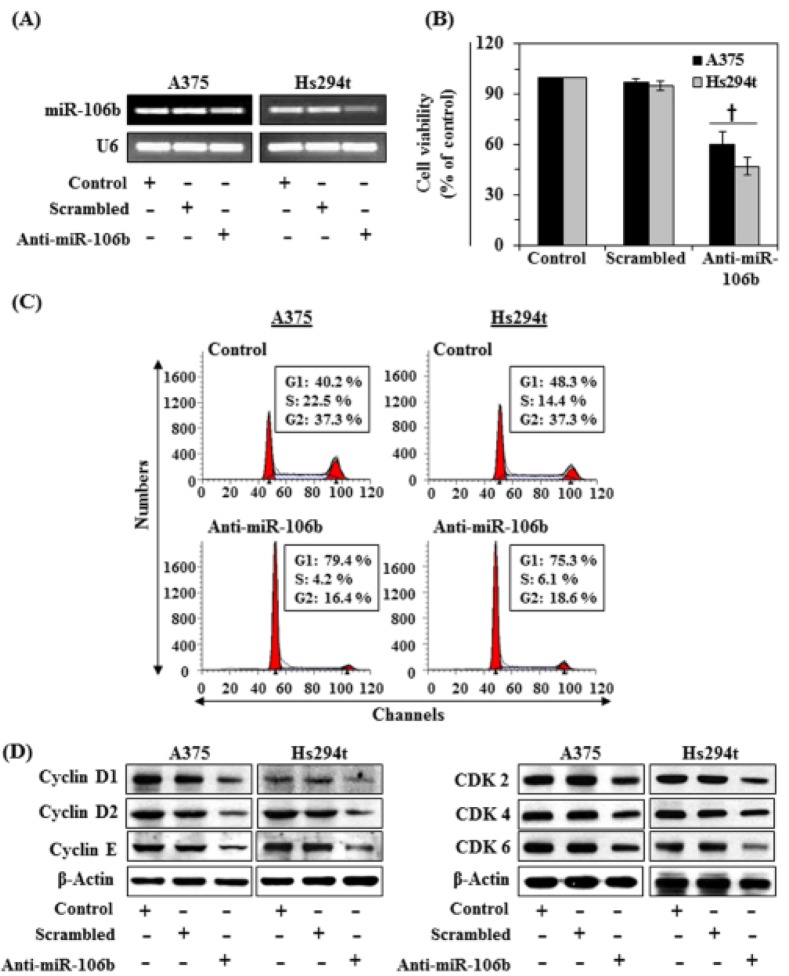
Suppression of miR-106b in melanoma cells leads to a reduction in cell viability and G1-phase arrest of cell cycle (A) Melanoma cell lines (A375 and Hs294t) were transfected with siRNA (Anti-miR-106b, 70 nM) for 48 h. After transfection, miRNA was isolated by Trizol method and the expression levels of miR-106b analyzed using RT-PCR, as detailed in Materials and Methods. (B) Cell viability was determined after suppression of miR-106b in melanoma cells and is presented in terms of percent of control. Significant difference between control *vs* Anti-miR-106b. ^†^*P*<0.004. (C) After transfection, cells were harvested and processed for cell cycle distribution analysis using flow cytometry. (D) Cell lysates from all treatment groups were subjected to western blot analysis. Inhibition of miR-106b reduced the levels of cell cycle regulatory proteins in both cell lines as compared to controls.

In order to better understand the role of miR-106b in the proliferation of melanoma cells, we selected two melanoma cells lines, A375 and Hs294t. The levels of miR-106b in A375 and Hs294t cell lines were suppressed through transfection with anti-miR-106b using lipofectamine as detailed in the Materials and Methods section. As shown in Figure 2A, this transfection strategy resulted in suppression of miR-106b levels in both cell lines as compared with those transfected with scrambled miR and others controls. We then determined the effect of suppression of miRNA-106b on the cell proliferation using an MTT assay. We found that downregulation of miR-106b in A375 and Hs294t cells resulted in significant inhibitory function on cell proliferation respectively by 40% and 53% (*P*<0.005) compared to untreated controls (Fig. 2B), which suggested a relationship between miRNA-106b and the cell proliferation capacity of melanoma cells.

### Suppression of miR-106b leads to G0/G1 cell cycle arrest and inhibition of cell cycle regulatory proteins in melanoma cell lines

Based on the above results, we determined whether inhibition of cell viability after the suppression of miRNA-106b in melanoma cells is associated with its reported effects on cell cycle regulation. For this purpose, A375 and Hs294t cells were treated with anti-miR-106b for 48 h. The cells were then harvested and subjected to cell cycle analysis. We found that the A375 cells were arrested into G0/G1 phase of cell cycle in anti-miR-106b treated group (79.4%, *P*<0.01) as compared to the cells of the control group (40.2%), as shown in Figure 2C. Similar results were obtained on analysis of cell cycle progression in Hs294t cells. Cell division relies on the activation of cyclins, which bind to CDKs to induce cell-cycle progression towards S phase and, later, to initiate mitosis. Therefore, we checked the effect of G0/G1 cell cycle arrest on the regulatory proteins of this phase using western blot analysis. As shown in Figure 2D, suppression of miR-106b in A375 and Hs294t human melanoma cells caused inhibition of cyclin D1, D2 and E, and reduction in the expression levels of CDK2, CDK4 and CDK6 proteins in both cell lines. Thus, it can be concluded that in melanoma cells overexpression of miR-106b may have a role in enhanced cell cycle progression while downregulation of miRNA-106b is associated with arrest of the G0/G1 phase and suppression of the levels of cyclins and CDKs proteins associated with the G0/G1 phase of the cell cycle.

### Downregulation of miR-106b in melanoma cells after treatment with GSPs leads to reduction in the viability of melanoma cells

**Figure 3:**
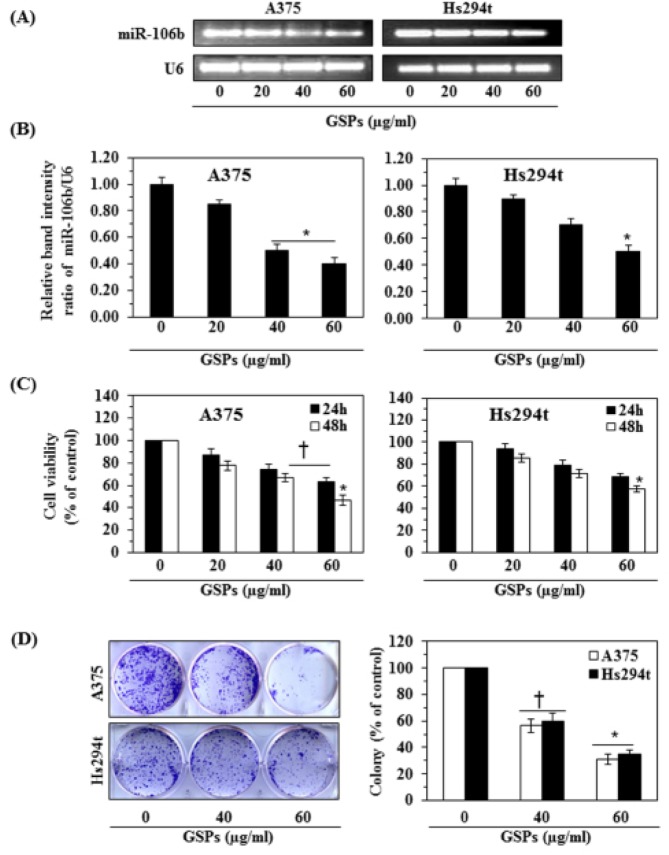
Effect of GSPs on miR-106b expression and cell viability in A375 and Hs294t melanoma cell lines *in vitro* (A) A375 and Hs294t cells were treated with various concentrations of GSPs (0, 20, 40, and 60 μg/ml) for 48 h. miRNA was isolated and subjected to miR-106b analysis using RT-PCR. Treatment of cells with GSPs reduced the expression levels of miR-106b. (B) Relative band intensity of miR-106b expression in melanoma cell lines after treatment of cells with GSPs. (C) Effect of GSPs on viability of melanoma cells after treatment for 24 h or 48 h. Data are presented in terms of percent of control as mean ± SD, n=6. (D) Effect of GSPs on the colony forming ability of melanoma cells. Colonies were detected after staining with crystal violet and photographed. Colonies appear dark blue-violet, and were counted using an Olympus microscope equipped with CellSens software. Data are summarized in terms of number of colonies/treatment group. A group of >50 cells was considered as one colony. Significant difference *versus* control, ^*^P<0.001; ^†^*P*<0.01.

We next tested whether GSPs have the ability to inhibit the over-expression of miRNA-106b in melanoma cells. The A375 and Hs294t cells were treated with various concentrations of GSPs (0, 20, 40 and 60 μg/ml) for 48 h. The cells were then harvested and the levels of miRNA-106b were analyzed using RT-PCR. The RT-PCR analysis revealed that treatment of melanoma cells with GSPs decreased the levels of miR-106b in a dose-dependent manner (Fig. 3A). The data for the two cell lines are summarized and presented in terms of the relative band intensity ratio of miRNA-106b *vs.* U6 in Figure 3B. The expression level of miRNA-106b was significantly reduced (*P*<0.01) after the treatment of these melanoma cell lines with GSPs.

As treatment with GSPs downregulated miR-106b expression in melanoma cells and suppression of miRNA-106b reduced the viability of melanoma cells, we further determined the effect of GSPs on the viability of melanoma cells. For this purpose, A375 and Hs294t melanoma cells were treated with different concentrations of GSPs for 24 and 48 h and cell viability was determined using an MTT assay. As shown in Figure 3C (left panel), treatment of A375 cells with GSPs reduced cell viability of cells in a dose-dependent manner (*P*<0.01) with the reduction in viability ranging from 13% to 37% (*P*<0.05) after 24 h, and 22% to 53% (*P*<0.01) after 48 h of treatment. Under identical conditions, a more or less similar pattern of GSPs-induced inhibitory effects were observed on Hs294t cells (Fig. 3C, right panel). We also verified the cytotoxic or anti-carcinogenic effects of GSPs on melanoma cells using colony formation assays, as detailed under Materials and Methods. We found that treatment of A375 and Hs294t cells with GSPs resulted in a reduction in the colony formation potential of the melanoma cells in terms of both the numbers of colonies and the size of the colonies, as shown in Figure 3D (left panel). GSPs treatment suppressed the colony formation ability of A375 cells by 45% and 69% at the doses of 40 and 60 μg/ml respectively, and suppressed the colony formation ability of Hs294t cells by 40% and 65% at the doses of 40 and 60 μg/ml respectively (Fig. 3D, right panel). In these experiments, colony formation data were analyzed in terms of percent of control (non-GSPs-treated group).

### GSPs induce G1-phase cell cycle arrest in melanoma cells

**Figure 4:**
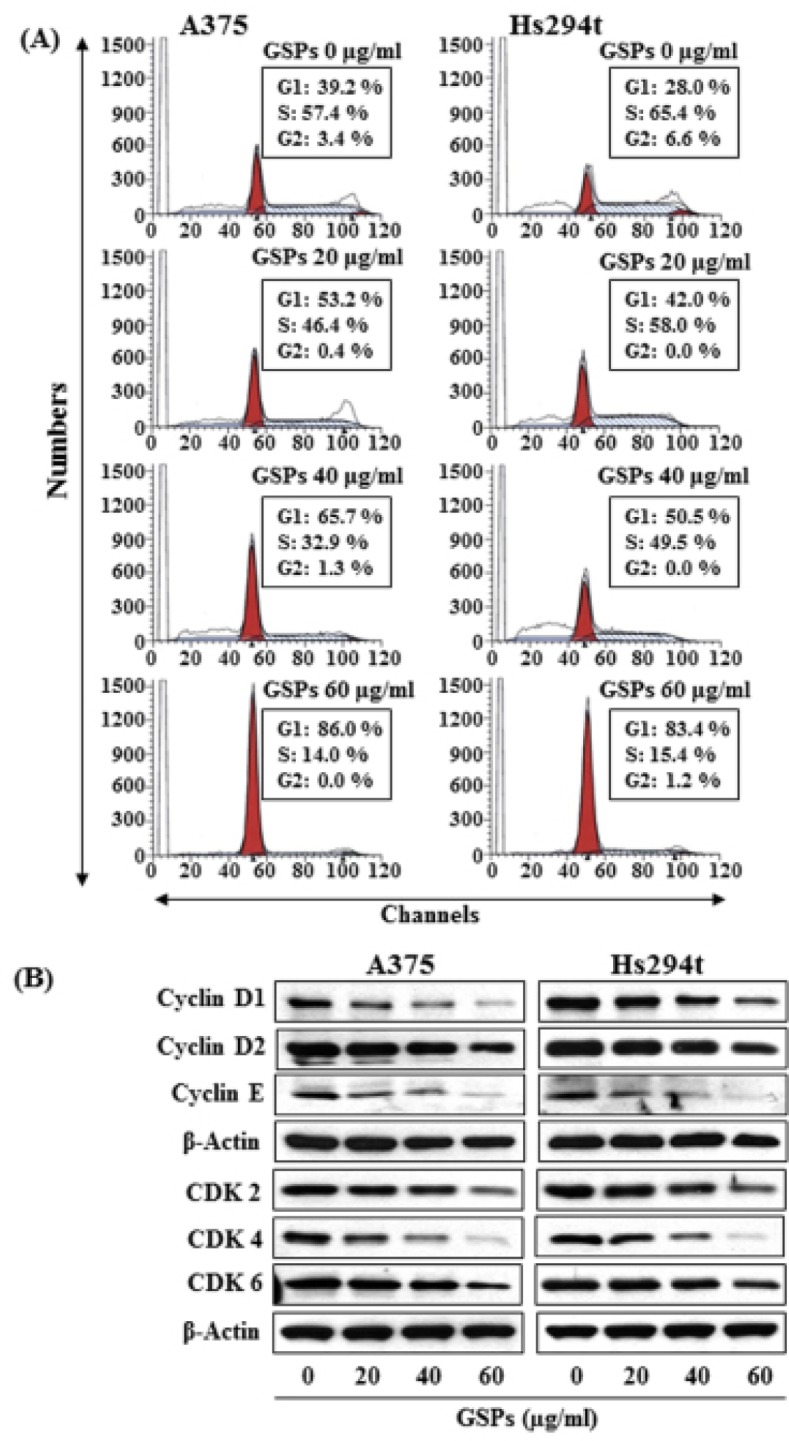
Effect of GSPs on cell cycle progression in melanoma cell lines (A) A375 and Hs294t cells were treated with or without GSPs for 48 h. After 48 h of treatment, cells were harvested and processed for cell cycle distribution analysis using flow cytometry. (B) Cell lysates were subjected to the analysis of cell cycle regulatory proteins of G1-phase using western blot analysis. GSPs inhibited the levels of cyclins and CDKs in melanoma cells as compared to non-GSPs-treated control cells in a dose-dependent manner. Equal protein loading on the gels was verified using antibody against β-actin.

Based on the effects of GSPs on cell viability, we selected doses of 20, 40 and 60 μg/ml of GSPs for further studies of cell cycle regulation in melanoma cells. As we have found that inhibition of miRNA-106b in melanoma cells resulted in G1-phase arrest (Fig. 2C), we determined whether inhibition of melanoma cell viability by GSPs also results in G1-phase cell cycle arrest. A375 and Hs294t cells were treated with GSPs for 48 h and cell cycle analysis was performed using FACS analysis, as described previously [23, 25]. As shown in Figure 4A, treatment of Hs294t cells with GSPs for 48 h resulted in an accumulation of a higher percentage of cells in the G1-phase of cell cycle in a dose-dependent manner: 20 μg/ml (42.0%), 40 μg/ml (50.5%, *P*<0.01) and 60 μg/ml (83.4%, *P*<0.001) as compared to the non-GSPs-treated controls (28.0%). It is important to note that the population in G1 phase of control cells (non-GSPs-treated) in Hs294t cells is less (28.0%, Fig. 4A) than the G1 phase cell population in control Hs294t cells of Fig. 2 (48%). This difference in G1 arrest population might be due to the difference in the number of passages of Hs294t cells and other experimental conditions. More or less similar patterns were found on analysis of the effects of GSPs treatment on cell cycle progression of A375 cells. These cell cycle data suggest that the GSPs-induced reduction in cell proliferation and cell viability in melanoma cells may be associated with the induction of G1 arrest by the GSPs, and that these changes may also be associated with the downregulation of miR-106b levels in melanoma cells on GSPs treatment.

### GSPs downregulate the levels of G1-phase linked cyclins and CDKs in melanoma cells

Based on the above data, we determined the effects of GSPs on cell cycle regulatory proteins in melanoma cells. The melanoma cells were treated with GSPs for 48 h and cell lysates were subjected to analysis of cell cycle regulatory proteins using western blot analysis. This revealed that treatment of A375 and Hs294t cells with GSPs for 48 h resulted in a reduction in the expression of cyclins D1, D2 and E in a dose-dependent manner (Fig. 4B). Similarly, a pronounced reduction in the expression levels of CDK2, CDK4 and CDK6 was observed in both A375 and Hs294t cell lines (Fig. 4B).

### P21/WAF1/Cip1/p21 is a direct target of miR-106b

**Figure 5:**
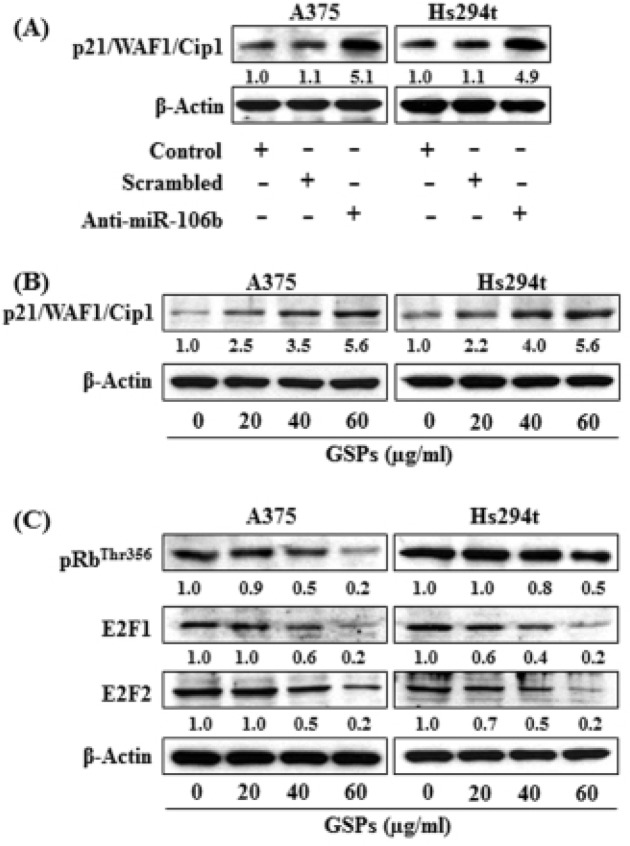
(A) Knockdown of miR-106b in A375 and Hs294t cells increased the expression of p21/WAF1/Cip1 protein in melanoma cells (B ' C) GSPs affect the p21/WAF1/Cip1/RB pathway in melanoma cells. Treatment of melanoma cells with GSPs for 48 h restored or enhanced the levels of p21/WAF1/Cip1 protein (B), and reduced the levels of E2F1 and E2F2 proteins (C) in a dose-dependent manner, as analyzed by western blot analysis. The relative density of each band in an immune-blot was analyzed using the ImageJ software (National Institute of Health). The numerical values are shown under each blot. For this purpose the band density of control group was arbitrarily selected as ‘1′ and comparison was then made with densitometry values of other treatment groups.

To verify whether tumor suppressor protein (p21/WAF1/Cip1) is the direct target of miR-106b, we treated the cells with anti-miR-106b and scrambled miR-106b for 48 h. Treatment of cells with anti-miR-106b decreased the levels of miR-106b (Fig. 2A) while enhancing or reactivating the levels of p21/WAF1/Cip1 in both A375 and Hs294t cells as compared to the control cells that were not treated with anti-miR-106b or treated with scrambled miRNA, as shown in Figure 5A.

### GSPs reactivate the expression of p21/WAF1/Cip1 protein

As we had found that treatment of melanoma cells with GSPs resulted in suppression of miR-106b (Fig. 3A ' 3B), we further determined whether treatment of GSPs upregulate or reactivate the expression of p21/WAF1/Cip1 in melanoma cells. Western blot analysis revealed that GSPs treatment reactivated or restored the levels of p21/WAF1/Cip1 in both A375 and Hs294t cell lines as compared to the cells which were not treated with GSPs (Fig. 5B). Further, treatment of GSPs also reduced the levels of pRb^Thr356^, E2F1 and E2F2 proteins in melanoma cells which are the downstream targets of p21/WAF1/Cip1, and this effect of GSPs was dose-dependent (Fig. 5C).

### Dietary administration of GSPs inhibit tumor xenograft growth of A375 cells in athymic nude mice

**Figure 6:**
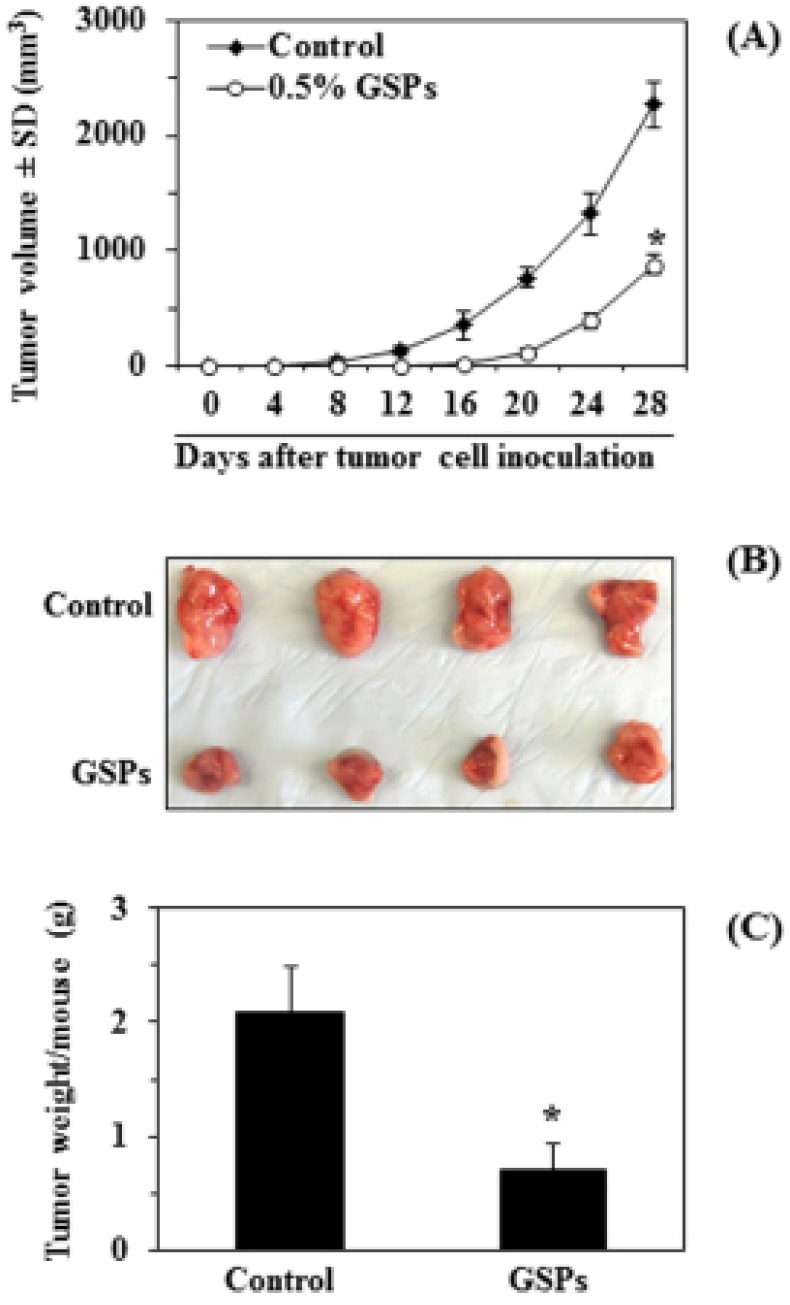
Dietary supplementation of GSPs with AIN76A control diet inhibits *in vivo* xenograft growth of A375 melanoma cells in athymic nude mice (A) Dietary administration of GSPs (0.5%, w/w) inhibited the growth of A375 cells grown as xenografts in athymic nude mice. Average tumor volume ± SD/mouse (mm^3^) in each group is reported as mean ±SD, n=8 per group. (B) The whole tumor mass was harvested from each mouse, photographed and is shown here for comparison. (C) Tumors were harvested at the termination of the experiment, and the wet weight of the tumor/mouse in grams is reported as the mean ± SD for each group. Statistical significance of difference between control and GSPs-treated groups was analyzed by one-way ANOVA, n=8/group. Statistical significance *vs.* non-GSPs-treated controls, ^*^P<0.001.

We further tested the effect of dietary GSPs on the growth of A375 tumor cell xenografts in immunocompromised athymic nude mice. As the effect of GSPs on A375 and Hs294t cells *in vitro* was almost identical, the *in vivo* tumor xenograft experiments were performed only with A375 melanoma cells. Based on our prior *in vivo* studies [23, 24], GSPs at a concentration of 0.5% were used to supplement the AIN76A control diet. To address the potential effect of GSPs on tumor xenograft growth of A375 cells, an equal number (4×10^6^) of A375 cells were injected subcutaneously into athymic nude mice and the growth of the tumor was recorded regularly as indicated in Figure 6A. Intake of dietary GSPs inhibited the growth of the A375 tumor xenografts throughout the experimental protocol, and at the termination of the experiment the inhibitory effect was 61% compared to the growth of tumor xenografts in mice fed the unsupplemented AIN76A diet (Fig. 6A). The inhibitory effect of GSPs on the growth of the tumor also was apparent in the visual appearance of the tumors harvested at the termination of the experiment as illustrated in Figure 6B. Further, at the termination of the experiment, the wet tumor weight (g)/mouse was determined for each mouse. As shown in Figure 6C, at the termination of the experiment, it was found that dietary GSPs significantly inhibited (66%, *P*<0.01) the growth of A375 tumor xenografts as compared to the growth of the xenograft tumors in non-GSPs-treated control mice.

### Dietary GSPs down regulates miR-106b expression in tumor xenograft tissues

**Figure 7:**
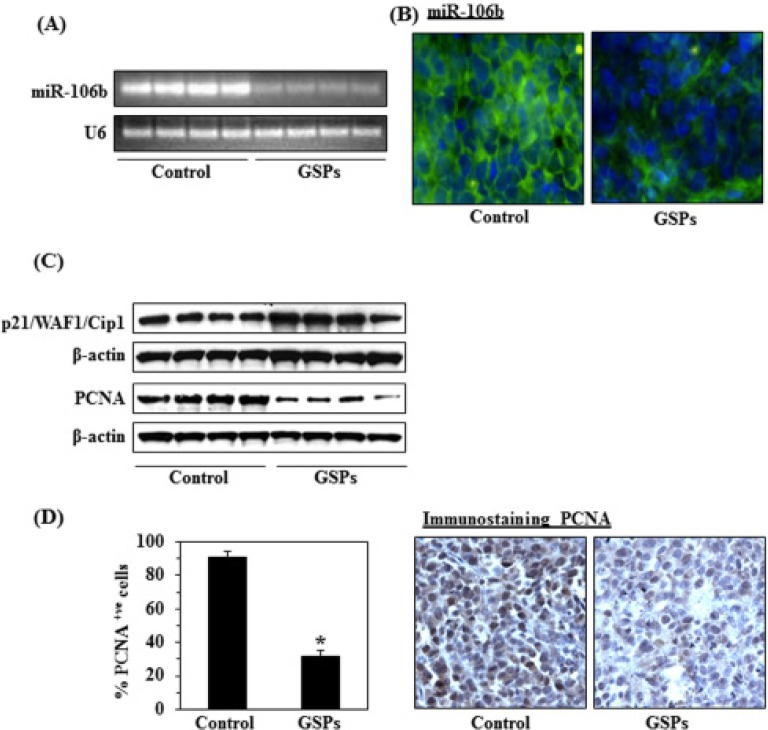
Dietary administration of GSPs (0.5%, w/w) altered the expression of miR-106b, p21/WAF1/Cip1 and PCNA in A375 tumor xenograft tissues (A) RT-PCR analysis of miR-106b expression in tumor samples from GSPs-fed and non-GSPs-fed control mice, n=4/group. (B) FISH detection of miR-106b in tumor xenograft tissues. miR-106b-positive *in situ* hybridization signals appear green, and DAPI nuclear stain appears blue, magnification x40. (C) Tumor cell lysates were subjected to western blot analysis of p21/WAF1/Cip1 and PCNA expression levels. (D) The immunohistochemical detection of PCNA-positive cells in tumor xenograft samples from GSPs-fed and non-GSPs-fed mice (Right panel). Resultant data on PCNA-positive cells are summarized (left panel). PCNA-positive cells are presented as the mean ± SD, n=4/group. Statistical significance *vs.* non-GSPs-treated control group of mice, ^*^P<0.001.

On RT-PCR analysis of miR-106b expression in the xenograft tumor tissues, we found that the expression level of miR-106b was markedly lower in the mice fed the GSPs-supplemented diet as compared with the control group (Fig. 7A). To further characterize the changes in miR-106b expression in the tumors, fluorescent in situ hybridization (FISH) was used to localize the expression pattern of miR-106b using Locked Nucleic Acid probe (Figure 7B). In concurrence with our RT-PCR data, *in situ* signals for miR-106b expression (shown in green) were very low in tumor sections obtained from mice fed GSPs as compared to the mice fed the control diet.

### GSPs reactivate p21/WAF1/Cip1 expression and inhibit proliferation potential of tumor cells in tumor xenografts

Our finding that downregulation of miR-106b restores the levels of p21/WAF1/Cip1 *in vitro* (Fig. 5B), suggested that p21/WAF1/Cip1 is a direct target of miR-106b. We therefore used western blot analysis to determine the expression levels of p21/WAF1/Cip1 in xenograft tumor tissues from GSPs-fed and control mice. As shown in Figure 7C, the results revealed that the expression level of p21/WAF1/Cip1 was increased or restored in tumor xenograft tissues from the mice that were fed GSPs as compared to the expression levels in tumor tissues from control group of mice which were not given GSPs in their diet. As uncontrolled tumor cell proliferation is a characteristic feature of most cancers, we also analyzed the A375 tumor xenografts for the potential antiproliferative effects of GSPs using western blot analysis of PCNA and immunohistochemical detection of PCNA-positive cells. The western blot analysis revealed that the expression level of PCNA was lower in tumor tissues obtained from GSPs-fed mice than in tumor tissues from control mice (Fig. 7C). The results of the immunohistochemical detection of PCNA-positive cells in tumor xenograft tissues confirmed that the percentage of proliferating cells was significantly lower (58%, *P*<0.01) in tumor xenografts from GSPs-treated mice than in the tumor xenografts from the control mice, as shown in Figure 7D.

## DISCUSSION

miRNA are small endogenous non-coding single-stranded RNAs that have the capability to interfere with the expression profile of several genes and proteins either by inducing a specific target mRNA or by reducing the translational capability of target mRNA [26]. miRNAs are involved in many pathologic and physiologic processes, including carcinogenesis. The role of miR-106b has been recognized in tumors of many organs [7, 8, 10-14]; however, little is known about its role in melanoma progression. In the present study, we analyzed the expression profile of miR-106b in seven different melanoma cell lines and NHEM using RT-PCR. Our study indicates that the expression of miR-106b is multi-fold higher (3-6 fold) in melanoma cells than in NHEM. miRNAs have been demonstrated to play important roles in various biological processes, such as cellular proliferation, oncogenesis, angiogenesis, and metastasis, and can act as oncogenes or tumor suppressors [27]. Our cell proliferation assay analysis indicates that the cell proliferation potential of melanoma cell lines (A375, Hs294t, SK Mel 28, SK Mel 119, Mel 1241, Mel 1011, and Mel 928) was several fold higher than that of NHEM and that this proliferation potential of melanoma cells is associated with the higher expression of miR-106b.

To verify whether overexpression of miR-106b in melanoma cells is associated with enhanced proliferation of cells, A375 and Hs294t cells were treated with an inhibitor of miRNA-106b and cell viability was determined. Downregulation of miR-106b resulted in suppression of melanoma cell viability, which suggests that the overexpression of miR-106b observed in the melanoma cells plays a key role in regulation of melanoma cell proliferation. This is consistent with the report that miR-106b is overexpressed in the majority of gliomas and that downregulation of miR-106b suppresses the growth of human glioma cells(28). Ivanovska *et al.* have shown that overexpression of miR-106b in cancer cells promotes cell cycle progression while downregulation inhibits it [16]. The authors also demonstrated that p21/WAF1/Cip1 is a direct target of miR-106b and its downregulation plays an effective role in miR-106b-induced cell cycle progression. Our cell cycle analysis showed that the treatment of melanoma cells (A375 and Hs294t) with anti-miR-106b (an inhibitor of miR-106b) markedly induces G1-phase arrest of both these cell lines indicating that the mechansim underlying the miR-106b-mediated upregulation of the proliferation potential of melanoma cells is associated with enhancement of cell cycle progression. Uncontrolled cell division or proliferation is dependent on the activation of cyclins and CDKs in G1-phase, which then interact and induce cell cycle progression towards S phase. CDK activity is one of the major causes of cancer progression. The functions of the CDKs are regulated by specific inhibitors, such as p21/WAF1/Cip1 [29]. p21/WAF1/Cip1 is generally overexpressed in response to anti-proliferative signals [30]. The G1 phase arrest in the melanoma cells after their treatment with anti-miR-106b was associated with marked suppression of the expression of both cyclins and CDKs (CDK2, CDK4 and CDK6) and concomitant reactivation of p21/WAF1/Cip1 protein. These results suggest that the ability of the inhibitor of miR-106b to block the uncontrolled cell cycle progression typical of melanoma cells and to induce their G1-phase arrest is mediated through suppression of the levels of cyclins and CDKs and reactivation of the tumor suppressor protein, p21/WAF1/Cip1. The ability of miRNAs to target multiple genes within a pathway is a well described phenomenon and suggests that therapeutic inhibition of these molecules may be extremely effective.

In efforts to develop an effective inhibitor of miR-106b for the treatment of melanoma, we tested the effect of GSPs on the expression level of miR-106b in melanoma cells. Our results suggested that treatment of melanoma cells with GSPs markedly lowered the levels of miR-106b in melanoma cells and that this resulted in a reduction in the viability and the colony forming ability of the cells. In similar *in vitro* experiments, the GSPs were found to reactivate the expression of p21/WAF1/Cip1, which may have played a crucial role in diminishing the carcinogenic potential of melanoma cells. Importantly, the levels of p21/WAF1/Cip1 also are overexpressed/reactivated in melanoma cells after treatment with anti-miR-106b. These data indicate two major observations: (i) GSPs act as an inhibitor of miR-106b in melanoma cells and (ii) that GSPs reactivate tumor suppressor protein p21/WAF1/Cip1 as does anti-miR-106b in melanoma cells. In addition to the effect of GSPs on p21/WAF1/Cip1, GSPs also affected the downstream signaling cascade of p21/WAF1/Cip1, as indicated by the inhibitory effects of GSPs on pRb, E2F1 and E2F2.

The *in vivo* studies conducted using immunocompromised athymic nude mice demonstrated that dietary GSPs exert a significant inhibitory effect on the growth of melanoma cell tumor xenografts and without apparent sign of toxicities in the mice. This inhibitory effect of GSPs on tumor xenograft growth was associated with the downregulation of miR-106b expression as well as upregulation of p21/WAF1/Cip1 protein, which results in suppression of tumor cell proliferation in the xenograft tissues.

In summary, we found that miR-106b is markedly upregulated in melanoma cells and acts as an oncogene by regulating the proliferation and cell cycle progression. In addition, our study reveals for the first time that GSPs have the ability to inhibit the proliferation of melanoma cells and block their cell cycle regulation through their inhibitory effect on miR-106b expression. Thus, our study suggests that (i) miR-106b might be a useful potential therapeutic target for melanoma treatment, and (ii) GSPs should be further investigated as a pharmacological agent alone or in combination with other therapeutic drugs for better management of melanoma in humans.

## MATERIALS AND METHODS

### Antibodies, chemicals and reagents

The antibodies specific for cyclins, pRb^Thr356^, E2F1, E2F2, CDK 2, CDK 4, CDK 6, p21/WAF1/Cip1, proliferating cell nuclear antigen (PCNA), β-Actin, and secondary antibodies horseradish peroxidase-linked anti-mouse IgG and anti-rabbit IgG were purchased from Santa Cruz Biotechnology (Santa Cruz, CA). Anti-miR-106b inhibitor, lipofectamine, primers specific for miRNA-106b and U6 were obtained from Invitrogen (Carlsband, CA).

### Cell lines and cell culture conditions

The human melanoma cells lines A375, Hs294t, and SK-Mel 28 were purchased from the American Type Culture Collection (Manassas, VA). Some other melanoma cell lines such as Mel 1241, Mel 1011, and Mel 928 were a kind gift from Dr. Paul Robbins (Center of Cancer Research, National Cancer Institute, Bethesda, MD). A375, Hs294t, Mel 1241, Mel 1011, and Mel 928 cell lines were cultured as monolayers in Dulbecco's modified Eagle's medium, whereas SK-Mel 119 and SK-Mel 28 were cultured in RPMI-1640 medium. The culture media were supplemented with 10% heat-inactivated fetal bovine serum (Hyclone, Logan, UT), 100 μg/ml penicillin and 100 μg/ml streptomycin and the cultures maintained in an incubator with 5% CO_2_ at 37^0^C. Normal human epidermal melanocytes (NHEMs) were obtained from the Cell Culture Core Facility of Skin Diseases Research Center at the University of Alabama at Birmingham, Birmingham, AL, and were cultured in HMGS supplemented melanocytes growth medium-254 (Life Technologies, Grand Island, NY).

For the treatment of cells, GSPs were dissolved in a small amount of DMSO (100 μl), which was added to the complete cell culture medium to attain the stipulated concentration of GSPs. The cells were treated when subconfluent (60-70%). The maximum concentration of DMSO in media was 0.1% (v/v).

### miRNA extraction and RT-PCR

Total RNAs, which contains 95% miRNAs, were isolated from cultured melanoma cell lines and normal human epidermal melanocytes using the TRIZOL-chloroform extraction procedure [31, 32]. Briefly, 70-80% confluent cultured cells were washed with ice-cold PBS buffer, the cells were covered with 1 ml Trizol (Sigma, St. Louis, MO) reagent and immediately harvested by scraping. The lysate was transferred into a 15 ml v-shaped tube and 0.2ml of chloroform was added for phase separation. The mixture was vortexed and centrifuged at 12,000 x g for 15 min at 4^°^C. After centrifugation, the uppermost colorless layer was separated into another tube and 5.0 ml of isopropyl alcohol was added for precipitation of RNAs. The sample was incubated at room temperature for 10 min and then centrifuged at 4^°^C. After centrifugation, the RNA pellet was washed with 75% ethanol then air dried and resuspended into nuclease-free water after which the RNA concentration was quantified by spectrophotometry. The RNA was used to prepare cDNA by using the iScript cDNA Synthesis Kit (Bio-RAD) according to the manufacturer's instructions. RT-PCR was performed using Platinum Taq DNA Polymerase (Invitrogen, Carlsbad, CA) with human specific primers for miR-106b: Forward primer: TAAAGTGCTGACAGTGCAGATAGTG, miR-106b Reverse primer: CAAGTACCCACAGTGCGGT, and U6 forward primer: CTCGCTTCGGCAGCACA, U 6 reverse primer: AACGCTTCACG AATTTGCGT, as reported previously [33]. The RT-PCR conditions were as follows: Stage I: 95°C for 3 min, 53°C for 1 min, 72°C for 30 sec (2 cycles); Stage II: 95°C for 3 min, 53°C for 1 min, 72°C for 30 sec (55 cycles); Stage III: 72°C for 5 min. The PCR product was run on a 2.5% agarose gel prepared in 1x Tris-acetate EDTA buffer containing ethidium bromide and analyzed using a Gel-Doc apparatus.

### Cell viability and colony formation assays

The viability of melanoma cell lines and NHEMs was determined using an MTT assay as described previously [23]. Briefly, 1×10^4^ cells were seeded in 96-well plates. After overnight incubation, the cells were treated with or without GSPs (0, 20, 40 and 60 μg/ml) for 24 and 48 h. At the end of the stipulated time, cells were treated with 50 μl of 5 mg/ml MTT and the resulting formazan crystals were dissolved in 150 μl of DMSO. The absorbance was recorded at 540 nm using a Bio-Rad 3350 microplate reader. The effect of GSPs on cell viability was calculated in terms of percent of control, which was arbitrarily assigned a value of 100% viability. We have also compared the proliferation capacity of melanoma cell lines without any treatment using MTT assay.

To assess the effects of GSPs on colony formation, melanoma cells suspended in complete medium were seeded in wells (2 × 10^3^ cells per well). After 4 d, the cells were treated with GSPs (40 and 60 μg/ml) for another 14 d. The cultures were maintained in a CO_2_ incubator during this time. The colonies were then stained with crystal violet and counted using an Olympus BX41 microscope fitted with the cellSens Software (Center Valley, PA).

### Cell cycle analysis

Cell cycle analysis was performed using flow cytometry with the use of propidium iodide staining (5 μg/ml), as described previously [24], after 48 h of GSPs treatment or miRNA transfection. Briefly, 1 × 10^6^ cells were trypsinized, washed with PBS, resuspended in chilled methanol, and kept at 4^°^C for 15 min. Cells were then centrifuged, washed in PBS, resuspended in 450 μl of PBS and 50 μl of RNase A (2 mg/ml), and incubated at 37^°^C for 30 min. Following RNase treatment, 500 μl of propidium iodide was added, and cells were incubated at room temperature for 60 min in the dark. Cell cycle analysis was performed using FACScaliber Flow Cytometer (BD Biosciences, San Jose, CA) equipped with Cell Quest 3.3 software in the Core Facility of the UAB Comprehensive Cancer Center. Analysis of cell cycle distribution was carried out using ModFit software (Verity Software House, Topsham, ME).

### Western blot analysis

Cell lysates were prepared following the treatment of melanoma cells for the indicated time periods, as detailed previously [25]. Tumor lysates were prepared similarly for the analysis of protein biomarkers. Proteins were resolved using 10-12% SDS-PAGE gels and transferred onto a nitrocellulose membrane. After blocking the non-specific binding sites, the membrane was incubated with the primary antibody overnight at 4^°^C. The membrane was then incubated with the appropriate peroxidase-conjugated secondary antibody. Specific protein bands were visualized using the enhanced chemiluminescence reagents. Equal loading of proteins on the gel was verified by stripping the membrane and re-probing with an anti-β-actin antibody.

### Transient transfection of miR-106b

For functional analysis, the expression of miR-106b in melanoma cells was silenced using a pre-designed anti-miR-106b inhibitor (Ambion, Austin, TX) following the manufacturer's instructions. Briefly, 1×10^5^ cells were seeded onto 6-well culture plates. A375 and Hs294t cell lines (60-70% confluent) were transfected in serum-free medium with the anti-miRNA inhibitor, or scramble control probe, at a final concentration of 70 nM, using Lipofectamine 2000 following the manufacturer's protocol (Invitrogen). After 24 h of transfection, cells were kept in a culture medium containing 2% FBS up to 48 h. The cells were then harvested and used in the functional assay, cell cycle distribution and western blot analysis. Both untransfected and scramble probe were used as controls for the transfected A375 and Hs294t cells.

### *In vivo* tumor xenograft study

Female athymic nude mice of 4- to 5-weeks of age were purchased from the National Cancer Institute (Bethesda, MD) and housed in the Animal Resource Facility at the University of Alabama at Birmingham in accordance with the Institutional Animal Care and Use Committee guidelines. The animal protocol used in this study was approved by the Institutional Animal Care and Use Committee (IACUC) of the University of Alabama at Birmingham. A375 melanoma cells (4x 10^6^ in 100 μl PBS/animal) were injected subcutaneously into the right flank of each mouse. After one day of cell inoculation, animals were divided randomly into two groups. One group of mice received the AIN76A control diet, while the second group of mice received a 0.5% GSPs-supplemented AIN76A control diet in pellet form throughout the experimental protocol. Each group has 8 mice. The experiment was terminated at the 4^th^ week after tumor cell inoculation. The tumor growth was recorded on a weekly basis, and tumor size was measured using Vernier calipers. Volumes were calculated using the hemiellipsoid model formula: tumor volume = ½ (4π/3) (l/2) (w/2) h, where h = height, w = width and l = length. At the termination of the experiment, mice were sacrificed and the tumor from each mouse excised. A portion of the tumor was used to isolate miRNAs, immunostaining and another part used to prepare tumor lysates for western blot analysis.

### Immunohistochemical detection of PCNA-positive cells

Paraffin-embedded tumor sections (5 μm thick) were deparaffinized, rehydrated and then an antigen retrieval procedure was carried out, as detailed previously [24]. Briefly, after blocking the non-specific binding sites, the sections were incubated with primary antibody for PCNA. After washing, the sections were incubated with biotinylated secondary antibody followed by horseradish peroxidase-conjugated streptavidin. The sections were further treated with 2,4-diaminobenzidine substrate and counterstained with hematoxylin. The PCNA-positive cells were counted in 3-4 different fields and photographed using an Olympus microscope (Model BX40F4, Tokyo, Japan) fitted with a Q-color 5 Olympus camera.

### Fluorescence in situ hybridization detection of miR-106b in tumor sections

For the detection of expression levels of miR-106b in tumor sections, a FISH assay was performed using the following LNA/DNA oligos sequences: LNA-miR-106b 5′-ATCTGCACTGTCAGCACTTTA-3′, scramble 5′-GTGTAACACGTCT ATACG CCCA-3′ [34]. Briefly, the formalin-fixed paraffin-embedded tissue sections were deparaffinized in xylene (2x 10 min) and rehydrated in serial ethanol solutions (100%, 95%, and 75%, v/v). The slides were then treated with 20 μg/ml proteinase K in TE buffer (100 mM Tris-Hcl, 50 mM EDTA, pH 8.0) for 10 min at 37°C, and fixed with 4% paraformaldehyde at 4°C. After blocking the endogenous peroxidases with 1% H_2_O_2_ for 30 min, slides were prehybridized in prehybridization buffer (4 x SSC containing 50% formamide) at the 37°C for 30 min. After prehybridization, slides were hybridized in hybridization buffer (40% formamide, 10% Dextran sulfate, 1 x Denhardt's solution, 4 x SSC, 10 mM DDT, 1 mg/ml yeast t-RNA, 1 mg/ml sheared salmon sperm DNA) with specific DIG-labeled probes at 50°C for 24 h. The sections were washed with gradient SSC thoroughly (2x SSC, 0.5X SSC and 0.2X SSC) to remove the background signals, followed by treatment with anti-digoxin FITC-conjugated antibody at 37°C for 2 h. Nuclei were counterstained with DAPI, and then sections were analyzed and imaged using a fluorescence microscope (Olympus, Japan) at 40x magnification using cellSens software.

### Statistical analysis

Student's *t*-test or one-way ANOVA was used to analyze cell viability, colony formation and tumor size using GraphPad Prism software (San Diego, CA). In each case, *P*<0.05 was considered statistically significant.
